# Editorial to special issue “The power of immunoprofiling supported by computational data integration and machine learning”

**DOI:** 10.1080/21645515.2024.2322327

**Published:** 2024-03-15

**Authors:** Elke Bergmann-Leitner

**Affiliations:** Biologics Research & Development, Center Infectious Disease Research, Walter Reed Army Institute of Research, Silver Spring, MD, USA

The immune system holds the key for well-being – and survival of the host – depending on how it responds to pathogens, tumor cells, and self. The innate and the adaptive immune systems collaborate to achieve this goal, and our knowledge regarding how these two arms of the immune system function continues to evolve as profiling studies of immune responses based on multi-omics approaches often lead to dogma-shifting discoveries. A high-profile example is the recognition that innate immune responses are not genomically imprinted and inflexible. Instead, exposure to pathogens or even vaccines result in trained immunity (“innate memory”) through mechanisms such as epigenetic changes in hematopoietic stem cells that profoundly alter the response patterns of innate immune cells to even antigenically unrelated subsequent pathogen encounters. Immunoprofiling has emerged as a powerful approach for deciphering immune reactions to diseases, infections, or therapeutics. The results are invaluable for (a) biomarker discovery by profiling immune cells and molecules and identify those indicative of presence, severity, and progression; (b) vaccine development; identifying correlates of protection, and optimize vaccine formulations to enhance immune responses and vaccine efficacy; (c) drug development; evaluating the effects of drugs and therapeutic agents on the immune system, researchers can assess drug safety, identify potential immune-related adverse effects, and optimize drug dosing regimens.

This special edition highlights a range of immunoprofiling studies, demonstrating the potential of this innovative approach. Additionally, it offers an overview of various computational methods utilized in the field and potential pitfalls and limitations. Here are the highlights of the various contributions:

## The role of immunoprofiling in informing agnostic treatments to boost immunity

Our understanding of factors impacting immunity including lifestyle, fitness, hygienic conditions, nutrition, substance abuse continues to advance. Many of these factors can profoundly alter the microbiome. We have only recently begun to appreciate how microbiota affect a variety of organ systems and their function, and thus overall health including mental health. In the context of immune health, the presence or even temporary absence (due to antibiotic treatment) of microbiota has a dramatic and long-lasting impact on the development of the immune system and immune function. Immunoprofiling contributes to the identification of factors involved in modulating immunity and can inform the design of immunotherapies aiming to boost immunity especially in populations with impaired immunoreactivities such the very young and geriatric individuals.

## The role of immunoprofiling in identifying immune mechanisms involved susceptibility vs. resilience against specific diseases

This special issue features a report by Wang et al.^1^ that showcases the establishment of a SARS-CoV2-specific immunoprofile and the computational analysis that revealed critical signaling pathways and immune signatures involved in COVID-19 morbidity. The data emphasize the role of copper-death related signaling pathways in the function of immune cells. This contribution exemplifies the necessity of conducting broad immunoprofiling campaigns, avoiding narrow hypotheses that may overlook new insights due to overly focused readouts.

## The role of immunoprofiling in vaccine design and identification of correlates of immunity

The design and evaluation of vaccine candidates in the absence of efficacy (challenge) studies continues to be hampered by the lack of known correlates of protection. Often, vaccine responses are evaluated based on a standard set of immunoassays such as determining antibody titers and measuring the frequency of polyfunctional T lymphocytes regardless of whether these responses contribute to protection against the respective pathogen. While such results do inform on the immunogenicity of vaccine formulations, they frequently fail to predict the protective efficacy of a vaccine. Even in the process of down-selecting adjuvants for a vaccine such readout methods are insufficient as discussed by Vázquez-Maldonado et al.^2^

Connors et al.^3^ expand the breadth of this special issue by reviewing various methodologies employed to profile immune responses and the computational approaches to establish immunological landscapes. Their discussion focuses on how computational tools can deduct confounding factors such as baseline responses and aging that impact innate immunity and ultimately vaccine efficacy.

In their research article Mura et al.^4^ report the identification of protective immune signatures mediated by vaccination with a whole parasite malaria vaccine (that is considered the gold standard for a vaccine able to confer sterile protection). This study also demonstrates the vastly improved sensitivity of transcriptomics when applied to antigen-specific cells that were sorted prior to analysis. Previous work by other groups failed to identify signatures associated with protection. However, those studies had been conducted using whole blood, which may otherwise have resulted in too much noise to detect antigen-specific responses. The results will be invaluable for next-gen malaria vaccine design and down-selection of vaccine candidates.

## Computational methodologies supporting immunoprofiling

Immunoprofiling generates high-density data sets that require computational integration and data analysis tools. This special issue provides an overview of the methodologies employed for data integration/analysis and machine learning and reports the use of immunoprofiling in various aspects of vaccine design.

In the methods portion of the special issue, Xiao et al.^5^ review the procedures and workflow of computational data integration and mining of omics data. The objective of integrating and mining omics data is to identify immune correlates of protection induced by vaccines and other immunotherapies. Zhang et al.^6^ review the statistical and artificial intelligence methods that are well suited for single cell analyses and with the goal of revealing biomarkers. As immunoprofiling and modeling of immune responses increasingly gain traction, concerns regarding the lack of reproducibility and repeatability of results are increasingly being raised. Gygi et al.^7^ address potential pitfalls of currently used models and how to avoid overfitting of models. Some issues leading to irreproducible modeling results or low predictive values arise from low sample sizes and lack of sufficiently high vaccine efficacy. Uncertainty about actual vaccine efficacy because perceived protected study participants may simply not have had exposure further complicates assessments. This latter point is addressed by Kelkar et al.^8^ The authors detail their approach to simulating immunological responses in vaccine efficacy trials with special consideration for the fact that participants without morbidity could either be truly protected or had simply not been exposed to the pathogen. The developed method evolved from evaluating various machine learning approaches and data simulations. Adding the probability of exposure enhances the power of this approach and may be an invaluable tool when designing vaccine efficacy trials.

## Disclaimer

Material has been reviewed by the Walter Reed Army Institute of Research. There is no objection to its presentation and/or publication. The opinions or assertions contained herein are the private views of the authors, and are not to be construed as official, or as reflecting the views of the Department of the Army or the Department of Defense. This paper has been approved for public release with unlimited distribution.

## References

[1] Wang Q, Su Z, Zhang J, Yan H, Zhang J. Unraveling the copper-death connection: decoding COVID-19‘s Immune landscape through advanced bioinformatics and machine learning approaches. Hum Vaccin Immunother. 2024. doi:10.1080/21645515.2024.2310359. PMC10936617 38468184

[2] Vázquez-Maldonado N, Kelly HR, Leitner WW. Comprehensive immunoprofiling and systematic adjuvant comparisons for identifying suitable vaccine: adjuvant pairings. Hum Vaccin Immunother. 2023;19(2):2223503. doi:10.1080/21645515.2023.2223503. 37341528 PMC10286686

[3] Connors J, Cusimano G, Mege N, Woloszczuk K, Konopka E, Bell M, Joyner D, Marcy J, Tardif V, Kutzler MA, et al. Using the power of innate immunoprofiling to understand vaccine design, infection, and immunity. Hum Vaccin Immunother. 2023;19(3):2267295. doi:10.1080/21645515.2023.2267295. 37885158 PMC10760375

[4] Mura M, Misganaw B, Gautam A, Robinson T, Chaudhury S, Bansal N, Martins AJ, Tsang J, Hammamieh R, Bergmann-Leitner E. Human transcriptional signature of protection after Plasmodium falciparum immunization and infectious challenge via mosquito bites. Hum Vaccin Immunother. 2023;19(3):2282693. doi:10.1080/21645515.2023.2282693. 38010150 PMC10760396

[5] Xiao H, Rosen A, Chhibbar P, Moise L, Das J. From bench to bedside via bytes: multi-omic immunoprofiling and integration using machine learning and network approaches. Hum Vaccin Immunother. 2023;19(3):2282803. doi:10.1080/21645515.2023.2282803. 38100557 PMC10730168

[6] Zhang J, Li J, Lin L. Statistical and machine learning methods for immunoprofiling based on single-cell data. Hum Vaccin Immunother. 2023;19(2):2234792. doi:10.1080/21645515.2023.2234792. 37485833 PMC10373621

[7] Gygi JP, Kleinstein SH, Guan L. Predictive overfitting in immunological applications: pitfalls and solutions. Hum Vaccin Immunother. 2023;19(2):2251830. doi:10.1080/21645515.2023.2251830. 37697867 PMC10498807

[8] Kelkar NS, Morrison KS, Ackerman ME. Foundations for improved vaccine correlate of risk analysis using positive-unlabeled learning. Hum Vaccin Immunother. 2023;19(1):2204020. doi:10.1080/21645515.2023.2204020. 37133899 PMC10294723

